# 2-(2-Chloro­phen­yl)-*N*-cyclo­hexyl-2-oxoacetamide

**DOI:** 10.1107/S1600536813005904

**Published:** 2013-03-09

**Authors:** Xiu-Dan Jin, Jin-Long Wu

**Affiliations:** a Laboratory of Asymmetric Catalysis and Synthesis, Department of Chemistry, Zhejiang University, Hangzhou, Zhejiang 310027, People’s Republic of China

## Abstract

In the title compound, C_14_H_16_ClNO_2_, the cyclo­hexyl ring has a chair conformation. The dihedral angle between the benzene ring and the mean plane of the four planar C atoms of the cyclo­hexyl ring is 45.2 (3)°. The two carbonyl groups are *trans* to one another, with an O=C—C=O torsion angle of −137.1 (3)°. In the crystal, mol­ecules are linked by N—H⋯O hydrogen bonds forming chains propagating along [001]. A region of disordered electron density, situated near the unit-cell corners, was treated using the SQUEEZE routine in *PLATON* [Spek (2009 ▶). *Acta Cryst.* D**65**, 148–155]. It gave a solvent-accessible void of *ca* 400 Å^3^ for only 21 electrons. It is probably due to traces of the solvent of crystallization and was not taken into account during structure refinement.

## Related literature

For the crystal structures of substituted phenyl­glyoxamides, see: Boryczka *et al.* (1998 ▶); Dai & Wu (2011 ▶); Jia & Wu (2012 ▶).
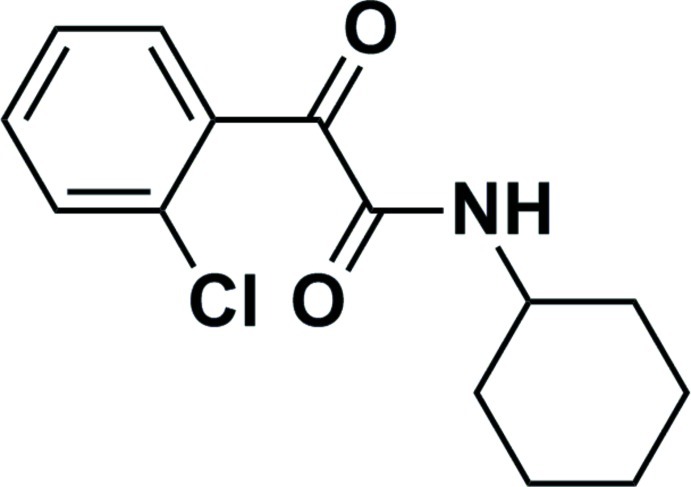


## Experimental

### 

#### Crystal data

C_14_H_16_ClNO_2_
*M* *_r_* = 265.73
Hexagonal, 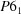

*a* = 17.075 (3) Å
*c* = 9.4536 (13) Å
*V* = 2387.0 (7) Å^3^
*Z* = 6
Mo *K*α radiation
μ = 0.24 mm^−1^
*T* = 293 K
0.48 × 0.26 × 0.20 mm

#### Data collection

Agilent Xcalibur (Atlas, Gemini ultra) diffractometer
Absorption correction: multi-scan (*CrysAlis PRO*; Agilent, 2011 ▶) *T*_min_ = 0.896, *T*_max_ = 0.955
15794 measured reflections
3101 independent reflections
2344 reflections with *I* > 2σ(*I*)
*R*_int_ = 0.047

#### Refinement

*R*[*F* ^2^ > 2σ(*F* ^2^)] = 0.051
*wR*(*F* ^2^) = 0.149
*S* = 1.01
3101 reflections
163 parameters
1 restraint
H-atom parameters constrained
Δρ_max_ = 0.19 e Å^−3^
Δρ_min_ = −0.17 e Å^−3^
Absolute structure: Flack (1983 ▶), 1422 Friedel pairs
Flack parameter: −0.05 (3)

### 

Data collection: *CrysAlis PRO* (Agilent, 2011 ▶); cell refinement: *CrysAlis PRO*; data reduction: *CrysAlis PRO*; program(s) used to solve structure: *SHELXS97* (Sheldrick, 2008 ▶); program(s) used to refine structure: *SHELXL97* (Sheldrick, 2008 ▶); molecular graphics: *OLEX2* (Dolomanov *et al.*, 2009 ▶); software used to prepare material for publication: *OLEX2*.

## Supplementary Material

Crystal structure: contains datablock(s) I, global. DOI: 10.1107/S1600536813005904/su2558sup1.cif

Structure factors: contains datablock(s) I. DOI: 10.1107/S1600536813005904/su2558Isup2.hkl

Supplementary material file. DOI: 10.1107/S1600536813005904/su2558Isup3.cml

Additional supplementary materials:  crystallographic information; 3D view; checkCIF report

## Figures and Tables

**Table 1:** Hydrogen-bond geometry (Å, °)

*D*—H⋯*A*	*D*—H	H⋯*A*	*D*⋯*A*	*D*—H⋯*A*
N1—H1⋯O2^i^	0.86	2.07	2.864 (3)	153

Symmetry code: (i) 
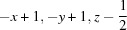
.

**Fig. 1.**
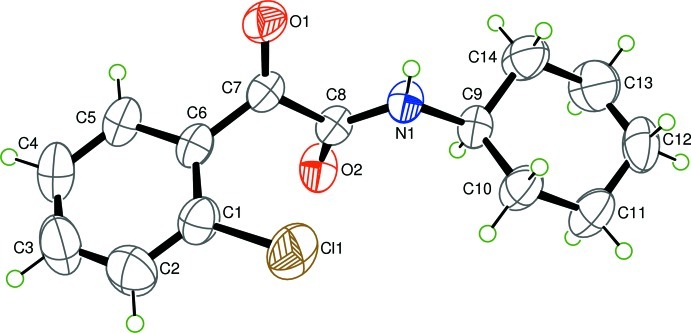
The molecular structure of the title molecule, with atom labelling. Displacement ellipsoids are drawn at 40% probability level.

**Fig. 2.**
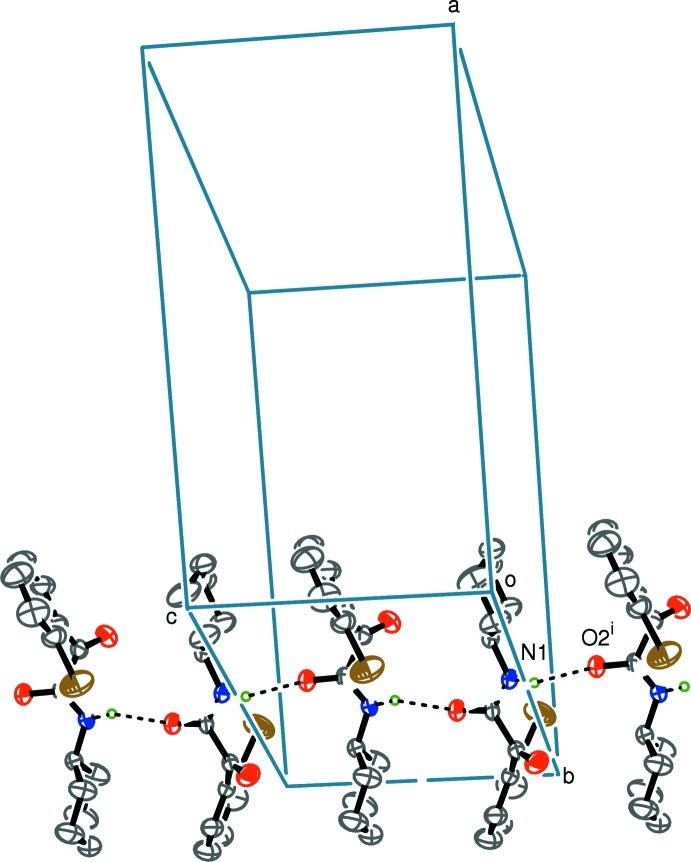
A view of the formation of the one-dimensional chain of the title compound via N—H···O hydrogen bonds [dashed lines; symmetry code: (i) -x+1, -y+1, z-1/2; see Table 1 for details].

## supplementary crystallographic information

### Comment

The crystal structure of several substituted phenylglyoxamides have been reported (Boryczka, *et al.*, 1998; Dai & Wu, 2011; Jia & Wu, 2012). The differences in their molecular packing depends on the hydrogen bonds present. In our effort to explore the effect of the substituent groups of phenylglyoxamide on the crystal form, we have synthesized the title compound by acetylation of cyclohexylamine with 2-chlorophenylglyoxyl chloride obtained from 2-chlorophenylglyoxic acid with oxalyl dichloride. We report herein on its crystal structure.

In the title molecule (Fig. 1), the cyclohexane ring has a chair conformation. The dihedral angle between the phenyl ring and the mean plane of the four planar C atoms of the cyclohexane ring (C10/C11/C13/C14) is 45.2 (3) °. The two carbonyl groups of the molecule are *trans* oriented to each other with a torsion angle O1═C7-C8 ═O2 of -137.1 (3) °.

In the crystal, molecules are linked by N—H···O hydrogen bonds forming chains extending in the c axis direction (Table 1 and Fig. 2).

### Experimental

To a solution of 2-chlorophenylglyoxylic acid (184 mg, 1.0 mmol) in dichloromethane (3 mL), was added oxalyl chloride (0.22 mL, 2.5 mmol) over 5 min. DMF (dimethylformamide) ( 1 drop) was then added and the solution was warmed to room temperature and stirred for 1.5 h. The solvent was removed under reduced pressure to afford 2-chlorophenylglyoxyl chloride which was used for the next step without further purification. To a solution of cyclohexylamine (0.23 mL, 2.0 mmol) and triethylamine (0.83 mL, 6.0 mmol) in dichloromethane (5 mL), was added dropwise the solution of the above glyoxyl chloride in dichloromethane (1 mL) at 273 K under N_2_, and the mixture was stirred for 4 h. The reaction was quenched with saturated NH_4_Cl solution (2 mL), then the organic layer was separated and the aqueous layer was extracted with dichloromethane (5 mL). The combined organic layers were washed with brine, dried over anhydrous Na_2_SO_4_ and filtered, the filtrate was concentrated under reduced pressure. The resulting residue was purified by column chromatography (silica gel, 20% ethyl acetate in hexane) to afford the title compound as colourless needles (227 mg, 85% yield from glyoxylic acid), m.p. 375-376 K. Single crystals suitable for X-ray diffraction were grown from a mixture of dichloromethane and hexane (1:1 v/v).

### Refinement

A region of disordered electron density, situated near the unit cell corners, was treated using the SQUEEZE routine in PLATON (Spek, 2009). It gave a solvent accessible void of ca. 400 Å^3^ for only 21 electrons. It is probably due to traces of the solvent of crystallization and was not taken into account during structure refinement.

The H atoms were placed in calculated positions and treated as riding atoms: N—H = 0.86 Å, C—H = 0.93, 0.98 and 0.97 Å for CH(aromatic), CH and CH_2_ atoms, respectively, with *U*_iso_(H) = 1.2*U*_eq_(N,C).

### Crystal data

**Table:**

C_14_H_16_ClNO_2_	*D*_x_ = 1.109 Mg m^−^^3^
*M**_r_* = 265.73	Mo *K*α radiation, λ = 0.71073 Å
Hexagonal, *P*6_1_	Cell parameters from 2933 reflections
Hall symbol: P 61	θ = 3.2–29.4°
*a* = 17.075 (3) Å	µ = 0.24 mm^−^^1^
*c* = 9.4536 (13) Å	*T* = 293 K
*V* = 2387.0 (7) Å^3^	Needle, colourless
*Z* = 6	0.48 × 0.26 × 0.20 mm
*F*(000) = 840	

### Data collection

**Table:**

Agilent Xcalibur (Atlas, Gemini ultra) diffractometer	3101 independent reflections
Radiation source: fine-focus sealed tube	2344 reflections with *I* > 2σ(*I*)
Graphite monochromator	*R*_int_ = 0.047
Detector resolution: 10.3592 pixels mm^-1^	θ_max_ = 26.1°, θ_min_ = 3.2°
ω scans	*h* = −16→21
Absorption correction: multi-scan (*CrysAlis PRO*; Agilent, 2011)	*k* = −21→19
*T*_min_ = 0.896, *T*_max_ = 0.955	*l* = −11→11
15794 measured reflections	

### Refinement

**Table:**

Refinement on *F*^2^	Secondary atom site location: difference Fourier map
Least-squares matrix: full	Hydrogen site location: inferred from neighbouring sites
*R*[*F*^2^ > 2σ(*F*^2^)] = 0.051	H-atom parameters constrained
*wR*(*F*^2^) = 0.149	*w* = 1/[σ^2^(*F*_o_^2^) + (0.0919*P*)^2^] where *P* = (*F*_o_^2^ + 2*F*_c_^2^)/3
*S* = 1.01	(Δ/σ)_max_ < 0.001
3101 reflections	Δρ_max_ = 0.19 e Å^−^^3^
163 parameters	Δρ_min_ = −0.17 e Å^−^^3^
1 restraint	Absolute structure: Flack (1983), 1422 Friedel pairs
Primary atom site location: structure-invariant direct methods	Flack parameter: −0.05 (3)

### Special details

**Table:**

Geometry. Bond distances, angles etc. have been calculated using the rounded fractional coordinates. All su's are estimated from the variances of the (full) variance-covariance matrix. The cell esds are taken into account in the estimation of distances, angles and torsion angles
Refinement. Refinement of F^2^ against ALL reflections. The weighted R-factor wR and goodness of fit S are based on F^2^, conventional R-factors R are based on F, with F set to zero for negative F^2^. The threshold expression of F^2^ > 2sigma(F^2^) is used only for calculating R-factors(gt) etc. and is not relevant to the choice of reflections for refinement. R-factors based on F^2^ are statistically about twice as large as those based on F, and R- factors based on ALL data will be even larger.

### Fractional atomic coordinates and isotropic or equivalent isotropic displacement parameters (Å^2^)

**Table:**

	*x*	*y*	*z*	*U*_iso_*/*U*_eq_	
Cl1	0.69837 (7)	0.67833 (8)	0.10734 (15)	0.1067 (4)	
O1	0.43847 (15)	0.60191 (15)	0.0815 (2)	0.0736 (8)	
O2	0.52877 (16)	0.55421 (14)	0.3612 (2)	0.0672 (8)	
N1	0.48258 (16)	0.46907 (14)	0.1620 (2)	0.0538 (8)	
C1	0.6611 (2)	0.7465 (2)	0.1859 (4)	0.0703 (11)	
C2	0.7225 (3)	0.8325 (3)	0.2271 (6)	0.1002 (16)	
C3	0.6930 (4)	0.8869 (3)	0.2830 (6)	0.1072 (18)	
C4	0.6038 (3)	0.8566 (2)	0.2982 (5)	0.0948 (16)	
C5	0.5408 (2)	0.7700 (2)	0.2549 (4)	0.0705 (11)	
C6	0.5693 (2)	0.71274 (18)	0.2003 (3)	0.0539 (9)	
C7	0.5009 (2)	0.61862 (19)	0.1599 (3)	0.0508 (9)	
C8	0.50637 (18)	0.54310 (18)	0.2353 (3)	0.0481 (8)	
C9	0.4826 (2)	0.38896 (18)	0.2192 (3)	0.0541 (9)	
C10	0.5628 (2)	0.3843 (2)	0.1677 (4)	0.0786 (14)	
C11	0.5633 (3)	0.3014 (3)	0.2262 (5)	0.0919 (17)	
C12	0.4763 (3)	0.2157 (2)	0.1922 (4)	0.0913 (16)	
C13	0.3966 (3)	0.2207 (3)	0.2424 (7)	0.112 (2)	
C14	0.3952 (2)	0.3033 (2)	0.1816 (6)	0.0897 (14)	
H1	0.46610	0.46740	0.07550	0.0650*	
H2	0.78410	0.85380	0.21720	0.1210*	
H3	0.73480	0.94550	0.31080	0.1280*	
H4	0.58450	0.89400	0.33780	0.1140*	
H5	0.47930	0.75020	0.26230	0.0840*	
H9	0.48640	0.39430	0.32250	0.0650*	
H10A	0.61760	0.43850	0.19660	0.0940*	
H10B	0.56180	0.38200	0.06510	0.0940*	
H11A	0.61380	0.29800	0.18590	0.1100*	
H11B	0.57150	0.30720	0.32790	0.1100*	
H12A	0.47190	0.20590	0.09070	0.1100*	
H12B	0.47680	0.16480	0.23670	0.1100*	
H13A	0.39770	0.22400	0.34490	0.1350*	
H13B	0.34180	0.16620	0.21460	0.1350*	
H14A	0.38860	0.29780	0.07960	0.1080*	
H14B	0.34410	0.30640	0.21980	0.1080*	

### Atomic displacement parameters (Å^2^)

**Table:**

	*U*^11^	*U*^22^	*U*^33^	*U*^12^	*U*^13^	*U*^23^
Cl1	0.0872 (6)	0.1113 (7)	0.1394 (9)	0.0629 (6)	0.0331 (6)	−0.0112 (7)
O1	0.0885 (15)	0.0802 (14)	0.0662 (14)	0.0527 (12)	−0.0205 (12)	−0.0011 (11)
O2	0.1098 (16)	0.0723 (12)	0.0345 (11)	0.0567 (12)	−0.0018 (10)	−0.0010 (9)
N1	0.0815 (16)	0.0613 (13)	0.0317 (11)	0.0455 (13)	−0.0049 (10)	0.0001 (10)
C1	0.078 (2)	0.0719 (19)	0.0720 (19)	0.0458 (17)	0.0126 (16)	0.0028 (16)
C2	0.071 (2)	0.080 (2)	0.134 (4)	0.026 (2)	0.012 (2)	0.000 (2)
C3	0.118 (3)	0.059 (2)	0.131 (4)	0.034 (2)	0.011 (3)	−0.005 (2)
C4	0.125 (3)	0.065 (2)	0.107 (3)	0.057 (2)	0.017 (3)	−0.002 (2)
C5	0.085 (2)	0.0680 (19)	0.075 (2)	0.0507 (17)	0.0183 (18)	0.0131 (17)
C6	0.0733 (18)	0.0610 (16)	0.0410 (15)	0.0437 (15)	0.0088 (12)	0.0106 (12)
C7	0.0668 (16)	0.0663 (16)	0.0346 (13)	0.0448 (14)	0.0051 (12)	0.0015 (11)
C8	0.0634 (15)	0.0629 (15)	0.0294 (14)	0.0402 (13)	0.0065 (11)	0.0036 (11)
C9	0.0799 (18)	0.0569 (15)	0.0362 (13)	0.0422 (14)	0.0037 (12)	0.0053 (11)
C10	0.073 (2)	0.073 (2)	0.101 (3)	0.0449 (17)	0.0086 (18)	0.0180 (19)
C11	0.100 (3)	0.095 (3)	0.109 (3)	0.070 (2)	0.008 (2)	0.019 (2)
C12	0.145 (4)	0.070 (2)	0.078 (2)	0.068 (2)	−0.015 (2)	−0.0055 (19)
C13	0.096 (3)	0.064 (2)	0.161 (5)	0.028 (2)	0.001 (3)	0.040 (3)
C14	0.072 (2)	0.068 (2)	0.128 (3)	0.0342 (18)	0.003 (2)	0.026 (2)

### Geometric parameters (Å, º)

**Table:**

Cl1—C1	1.747 (4)	C12—C13	1.484 (8)
O1—C7	1.210 (4)	C13—C14	1.534 (6)
O2—C8	1.235 (3)	C2—H2	0.9300
N1—C8	1.315 (3)	C3—H3	0.9300
N1—C9	1.471 (4)	C4—H4	0.9300
N1—H1	0.8600	C5—H5	0.9300
C1—C6	1.380 (5)	C9—H9	0.9800
C1—C2	1.367 (6)	C10—H10A	0.9700
C2—C3	1.365 (8)	C10—H10B	0.9700
C3—C4	1.349 (9)	C11—H11A	0.9700
C4—C5	1.386 (5)	C11—H11B	0.9700
C5—C6	1.391 (5)	C12—H12A	0.9700
C6—C7	1.489 (4)	C12—H12B	0.9700
C7—C8	1.517 (4)	C13—H13A	0.9700
C9—C14	1.520 (5)	C13—H13B	0.9700
C9—C10	1.493 (5)	C14—H14A	0.9700
C10—C11	1.524 (6)	C14—H14B	0.9700
C11—C12	1.509 (6)		
			
C8—N1—C9	123.8 (2)	C5—C4—H4	120.00
C9—N1—H1	118.00	C4—C5—H5	120.00
C8—N1—H1	118.00	C6—C5—H5	120.00
Cl1—C1—C6	118.7 (2)	N1—C9—H9	108.00
Cl1—C1—C2	119.9 (3)	C10—C9—H9	108.00
C2—C1—C6	121.4 (4)	C14—C9—H9	108.00
C1—C2—C3	119.7 (5)	C9—C10—H10A	109.00
C2—C3—C4	120.7 (4)	C9—C10—H10B	109.00
C3—C4—C5	120.2 (4)	C11—C10—H10A	109.00
C4—C5—C6	120.1 (4)	C11—C10—H10B	109.00
C1—C6—C7	122.7 (3)	H10A—C10—H10B	108.00
C1—C6—C5	117.8 (3)	C10—C11—H11A	109.00
C5—C6—C7	119.5 (3)	C10—C11—H11B	109.00
C6—C7—C8	116.7 (3)	C12—C11—H11A	109.00
O1—C7—C8	120.6 (3)	C12—C11—H11B	109.00
O1—C7—C6	122.4 (3)	H11A—C11—H11B	108.00
O2—C8—N1	125.3 (3)	C11—C12—H12A	109.00
O2—C8—C7	117.9 (2)	C11—C12—H12B	109.00
N1—C8—C7	116.7 (2)	C13—C12—H12A	109.00
N1—C9—C10	110.9 (2)	C13—C12—H12B	109.00
C10—C9—C14	111.0 (3)	H12A—C12—H12B	108.00
N1—C9—C14	110.6 (3)	C12—C13—H13A	109.00
C9—C10—C11	111.3 (3)	C12—C13—H13B	109.00
C10—C11—C12	111.3 (4)	C14—C13—H13A	109.00
C11—C12—C13	111.3 (4)	C14—C13—H13B	109.00
C12—C13—C14	111.7 (4)	H13A—C13—H13B	108.00
C9—C14—C13	109.8 (4)	C9—C14—H14A	110.00
C1—C2—H2	120.00	C9—C14—H14B	110.00
C3—C2—H2	120.00	C13—C14—H14A	110.00
C2—C3—H3	120.00	C13—C14—H14B	110.00
C4—C3—H3	120.00	H14A—C14—H14B	108.00
C3—C4—H4	120.00		
			
C8—N1—C9—C14	133.5 (4)	C1—C6—C7—C8	59.6 (4)
C9—N1—C8—O2	−2.4 (5)	C5—C6—C7—O1	52.5 (4)
C9—N1—C8—C7	−179.1 (3)	C5—C6—C7—C8	−120.4 (3)
C8—N1—C9—C10	−103.0 (3)	O1—C7—C8—N1	39.9 (5)
C6—C1—C2—C3	−0.4 (7)	C6—C7—C8—O2	36.0 (4)
Cl1—C1—C6—C5	−176.0 (3)	C6—C7—C8—N1	−147.0 (3)
Cl1—C1—C6—C7	3.9 (4)	O1—C7—C8—O2	−137.1 (3)
Cl1—C1—C2—C3	177.3 (4)	N1—C9—C10—C11	−179.9 (3)
C2—C1—C6—C5	1.7 (6)	C14—C9—C10—C11	−56.5 (4)
C2—C1—C6—C7	−178.4 (4)	N1—C9—C14—C13	−179.9 (4)
C1—C2—C3—C4	0.1 (8)	C10—C9—C14—C13	56.6 (5)
C2—C3—C4—C5	−1.1 (8)	C9—C10—C11—C12	55.2 (4)
C3—C4—C5—C6	2.4 (6)	C10—C11—C12—C13	−54.8 (5)
C4—C5—C6—C7	177.4 (3)	C11—C12—C13—C14	55.9 (6)
C4—C5—C6—C1	−2.6 (5)	C12—C13—C14—C9	−56.5 (6)
C1—C6—C7—O1	−127.4 (4)		

### Hydrogen-bond geometry (Å, º)

**Table:**

*D*—H···*A*	*D*—H	H···*A*	*D*···*A*	*D*—H···*A*
N1—H1···O2^i^	0.86	2.07	2.864 (3)	153

Symmetry code: (i) −*x*+1, −*y*+1, *z*−1/2.
